# Identifying Biological Signatures of N-Acetylcysteine for Non-Suicidal Self-Injury in Adolescents and Young Adults

**DOI:** 10.20900/jpbs.20210007

**Published:** 2021-04-29

**Authors:** Siddhee A. Sahasrabudhe, Thanharat Silamongkol, Young Woo Park, Alanna Colette, Lynn E. Eberly, Bonnie Klimes-Dougan, Lisa D. Coles, James C. Cloyd, Gülin Öz, Bryon A. Mueller, Reena V. Kartha, Kathryn R. Cullen

**Affiliations:** 1 Center for Orphan Drug Research, Department of Experimental and Clinical Pharmacology, College of Pharmacy, University of Minnesota, Minneapolis, MN 55455, USA; 2 Department of Psychiatry and Behavioral Sciences, Medical School, University of Minnesota, Minneapolis, MN 55454, USA; 3 Center for Magnetic Resonance Research, Department of Radiology, University of Minnesota, Minneapolis, MN 55455, USA; 4 School of Public Health, Division of Biostatistics, University of Minnesota, Minneapolis, MN 55455, USA; 5 Department of Psychology, College of Liberal Arts, University of Minnesota, Minneapolis, MN 55455, USA

**Keywords:** N-acetylcysteine, non-suicidal self-injury, biomarkers, antioxidant, MRS

## Abstract

The prevalence of non-suicidal self-injury (NSSI) is high in adolescents and young adults. However, there is a paucity of evidence-based treatments to address this clinical problem. An open-label, pilot study in the target population showed that treatment with oral N-acetylcysteine (NAC), a widely available dietary supplement, was associated with reduction in NSSI frequency. In preparation for a biologically informed design of an efficacy trial, a critical preliminary step is to clarify NAC’s biological signatures, or measures of the mechanisms underlying its clinical effects. Toward that end, we propose a 2-stage project to investigate NAC’s biological signatures (changes in glutathione (GSH) and/or glutamate (Glu)) in women with NSSI. The first stage; a double-blind randomized placebo-controlled study will focus on identifying the optimal dose to achieve meaningful change in GSH and Glu during short-term (4 weeks) NAC treatment in 36 women aged 16–24 years with NSSI. Go/No-go criteria to determine if the study will progress to the second stage include pre-specified changes in brain and blood measures of GSH. Changes in the brain GSH are measured through magnetic resonance spectroscopy (MRS). The dose for the stage 2 will be selected based on the biological changes and the tolerability observed in the stage 1. The stage 2 will seek to replicate the biological signature findings in an 8-week trial in a new patient cohort, and examine the relationships among biological signatures, NAC pharmacokinetics and clinical response. This 2-stage project is unique as it unifies clinical psychiatric measurements, quantitative MRS and pharmacological approaches in the first placebo-controlled clinical trial of NAC in young women with NSSI.

**Trial registration:**

The stage 1 trial protocol has been registered on https://clinicaltrials.gov/ with ClinicalTrials.gov ID “NCT04005053” (Registered on 02 July 2019. Available from: https://clinicaltrials.gov/ct2/show/NCT04005053).

## INTRODUCTION

Non-suicidal self-injury (NSSI) is defined as the intentional act of damaging one’s own body tissues without the intent of suicide [1]. The age of onset of NSSI typically coincides with adolescence [2], an important period for brain development [3]. Adolescent NSSI has an international prevalence of 18% [4]. NSSI is associated with long-term consequences including persistent psychopathology and future suicide attempts [5–7]. The current treatment of choice is dialectic behavioral therapy [8], an intensive psychotherapy intervention that takes about one year to deliver and represents a scarce resource in most communities. There is no FDA-approved drug for the treatment of NSSI and given the current challenges in managing this disorder, a pharmacological intervention may add a significant value. With the enhanced neuroplasticity inherent to developmental periods, a pharmacologically informed treatment for adolescent NSSI could have lasting health benefits.

N-acetylcysteine (NAC), a nutritional supplement and a prescription drug for indications including acetaminophen overdose and lung diseases, has recently been studied as a potential treatment for patients with various neuropsychiatric disorders [9]. A key biological mechanism that may underlie NAC’s positive impact on mental health is its capacity to increase glutathione (GSH), the primary antioxidant in the brain, providing a neuroprotective effect against the toxicities associated with stress [9,10]. Oxidative stress mechanisms have been implicated in affective disorders [11]. In adolescents, NSSI behavior typically represents an attempt to self-regulate extreme levels of negative affect [12]. Adolescents with NSSI frequently report a history of severe adverse experiences [13,14] which themselves are known to have lasting adverse impacts on the central nervous system (CNS) including neuronal damage [15–18]. Despite the relevance of oxidative stress mechanisms to NSSI, no research to date has formally tested treatments targeting this system in adolescents with NSSI using appropriate methodology to confirm this potential biological signature. Since NSSI is a maladaptive, habitual behavior, it has conceptual overlap with other disorders along the impulsive/compulsive spectrum such as addiction, hair-pulling, and skin-picking. Previous studies have demonstrated that NAC may alleviate these problems [19–21]. Dysregulation within the glutamate (Glu) system has been implicated in these habit-based disorders [22]. NAC’s primary metabolite cysteine (CYS) gets converted to cystine, which then enters glial cells in exchange for Glu via the Glu-cystine antiporter. A potential biological signature for NAC is its modulation of the Glu-cystine antiporter, downregulating excessive Glu transmission and associated excitotoxicity [23].

While multiple placebo-controlled clinical trials have shown that oral NAC, typically at modest doses in the range of 1200 mg–3000 mg/day, produces significant clinical improvement in several psychiatric disorders [9], mixed findings and gaps in knowledge have prevented the translation to clinical practice. Until recently, studies examining the effects of NAC on mental health or behavioral outcomes have generally not included explorations of pharmacokinetics (PK) and empirical pharmacodynamic (PD) responses representing a missed opportunity to explain mixed findings with respect to clinical efficacy. Therefore, previous failures in finding a significant effect of NAC on the improvement of neuropsychiatric outcomes may have stemmed from sub-optimal NAC levels in the blood and CNS. We still lack knowledge about the key neurometabolite changes that underlie NAC’s positive effects in NSSI. Newer studies, including our study, however; have included pharmacological aspects in addition to the clinical psychiatric evaluations to fill this gap [24,25]. Based on existing knowledge, there are at least two candidate biomarkers or biological signatures of NAC action: (1) increasing brain concentrations of GSH, the brain’s primary antioxidant, and (2) modulating Glu, serving to reduce stress-induced excitotoxicity. Both of these mechanisms are relevant to NSSI in that affected adolescents report that their NSSI episodes are triggered by past and repeated stressors. In this population it is likely that chronic stress takes a toll on the brain via oxidative stress and excitotoxic mechanisms [26]. Therefore, the potential of NAC to alleviate stress-induced oxidative damage and Glu-mediated neurotoxicity, can be beneficial in treating adolescents with NSSI. Research from our group has previously demonstrated a trend toward increased GSH levels as measured by magnetic resonance spectroscopy (MRS) following oral and IV NAC in adults with other diseases likely affected by oxidative stress [27,28]. Others have shown that single-dose NAC can lead to reduction in Glu levels in the anterior cingulate cortex (ACC) in adults with cocaine use disorder [29]. A double-bind, randomized, placebo-controlled study of oral NAC in patients with early psychosis investigated if brain GSH, blood cell GSH and GSH peroxidase activity could serve as biomarkers to guide the NAC treatment. Negative or positive symptoms and neurocognition in terms of cognitive speed served as clinical outcome measures. That study reported an increase in GSH levels in the medial prefrontal cortex, indicating target engagement [30]. There are many psychiatric studies that have investigated clinical, peripheral and cortical effects of NAC which are either completed or ongoing. Several of these studies have quantitatively explored blood or cortical GSH levels [24,25,31], some have included other peripheral measures of oxidative stress [24,25,32] and inflammation [24,32]. However, these candidate NAC mechanisms have not yet been studied in youth with NSSI.

In a previous open-label study, our group reported that adolescent girls showed a decrease in NSSI frequency after 8 weeks of oral NAC [33]. Additionally, we documented the feasibility of employing neuroimaging as an assessment tool in the context of the open-label NAC trial [33]. Interpretation of this clinical trial, however, is limited owing to the lack of placebo control group and the open label nature of the study. Important questions such as the mechanism of NAC action relevant to NSSI still remain to be answered. Next steps in investigating NAC as a potential treatment will require a clear understanding of NAC’s biological targets and dose-response or exposure-response relationships.

In the proposed study, we aim to address aforementioned knowledge gaps. The study is funded by National Center for Complementary and Integrative Health (NCCIH) through a two-step R61/R33 mechanism to investigate NAC’s biological signatures in adolescent NSSI. The first stage (R61) will focus on identifying the optimal dose of NAC to achieve meaningful change in GSH and Glu in the ACC as measured by MRS during short-term (4-week) treatment. For MRS assessment of neurochemical concentrations, we focus on the ACC, which is a key brain region implicated in emotion regulation and behavioral control. In addition, we will measure anatomical and functional characteristics of the brain using magnetic resonance imaging (MRI) and resting-state functional MRI (rs-fMRI). Although NSSI affects both genders, to reduce heterogeneity (e.g., due to sex differences in brain development) in a relatively small sample, this study focuses on adolescent females, as they have a relatively higher prevalence of NSSI [34,35].

Specific go/no go criteria have been established to determine whether the study will transition to the next stage (stage 2 i.e., R33). The dose for the stage 2 will be selected based on the biological changes and the tolerability observed in the stage 1. The stage 2 will seek to replicate the biological signature findings in an independent cohort using chronic (8-week) dosing and will examine the relationships among biological signatures, NAC PK, and clinical response.

Both stages R61 and R33 will be randomized placebo-controlled clinical trials examining the impact of the study drug on mental health outcomes. Assessments will include mental health questionnaires, PK measurement, the use of ultrahigh-field 7 tesla (7T) magnetic resonance (MR) technology which allows for reliable measurements of brain Glu and GSH with MRS, and a multimodal approach that also includes assessments of oxidative stress.

## HYPOTHESIS AND SPECIFIC AIMS

### Aim 1 (Stage 1, R61)

Measure effects on brain GSH, Glu and blood GSH redox ratio (GSH/GSSG) in 36 adolescent and young adult women with NSSI following a four-week regimen of oral NAC 5400 mg/day (*n* = 12), NAC 3600 mg/day (*n* = 12) or placebo (PBO) (*n* = 12).

#### Hypothesis 1a.

NAC but not PBO will produce dose-dependent improvements in blood GSH/GSSG and in cortical GSH concentrations, and dose-dependent decreases in cortical Glu concentrations.

#### Hypothesis 1b.

Greater blood/plasma levels of NAC and its metabolites will correlate with greater changes in biological signatures (e.g., greater increases in brain GSH, blood GSH/GSSG, but greater decreases in brain Glu).

### Aim 2 (Stage 2, R33)

Measure change in biological signatures and NSSI frequency in a new sample of 60 adolescent and young adult women with NSSI during an 8-week course of NAC (*n* = 30) versus PBO (*n* = 30) (dose selected based on the stage 1 results).

#### Hypothesis 2a.

Biological signatures as demonstrated in the stage 1 will be replicated in the context of the longer treatment course.

#### Hypothesis 2b.

Decrease in NSSI frequency will correlate with changes in biological signatures.

#### Hypothesis 2c.

Both extent of change in biological signatures and reduction in NSSI events will correlate with NAC/metabolite exposure.

## INNOVATION

Several innovative features of this 2-stage R61/R33 study underscore its promise for moving the field forward. First, to our knowledge, this is the first study to test NAC versus PBO in youth with NSSI. At present, there are no medications available known to alleviate this highly problematic behavior in youth. Thus, the work takes an initial step to address a significant gap. Second, the focus of this initial study is in establishing target engagement by NAC of proposed biological signatures (oxidative stress and on glutamate systems), including a multiple dosing strategy and PK analyses to allow examination of dose-response effects with respect to biological signatures. This multi-modal, target engagement approach in a 2-stage study allows for the examination of biological mechanisms (especially focusing on the CNS) in the context of a clinical trial, representing a critical step forward. Third, the use of state-of-the-art MRS acquisition using 7T MR, and a highly optimized protocol that is capable of collecting high-quality MRS data represents a key innovation that will allow us to reliably quantify Glu (separate from glutamine, something that is challenging on standard 3T systems) and GSH.

## MATERIALS AND METHODS

### Stage 1 (R61)

#### Study design

This is a randomized, double-blind, placebo-controlled study with two NAC doses to test whether a meaningful change in measurable biomarkers can be achieved following a 4-week course of NAC treatment. As there is no standard of care established for the treatment of NSSI, we aim to compare the effects of NAC against PBO. We are recruiting 36 girls and women aged 16–24 years who are assigned to one of 3 groups: low-dose NAC (3600 mg/day), high-dose NAC (5400 mg/day), or PBO (1:1:1). Because this is a small study, a baseline adaptive randomization procedure called minimization (rather than stratification) is used to provide balance across the treatment groups for each of 4 potentially confounding baseline factors: age (<20 vs 20+ years), Beck Depression Inventory score (BDI) (<29 vs 29+), NSSI frequency (<1/week vs 1+/week), and current psychotropic medication use (yes vs no). This design will allow us to confirm acute biological changes, select the optimal dose for achieving these effects that can be studied further in the next stage (stage 2), and examine dose/concentration-response relationships with respect to biological markers and PK.

#### Duration

Total study duration: 4 weeks of treatment.

#### Setting

This is a single-site study taking place at the University of Minnesota (UMN) psychiatric clinic. Clinical assessments will be conducted through the Psychiatry Department by a team of clinicians and clinical trainees. Neuroimaging procedures will be conducted at the Center for Magnetic Resonance Research (CMRR) while blood processing and analysis of NAC, NAC metabolites and other related biomarkers will be conducted at the Center for Orphan Drug Research (CODR), both at the UMN.

#### Recruitment

Participants are recruited through UMN Department of Psychiatry and Behavioral Sciences research registry, as well as the UMN campus and broader community through clinical referrals, flyers and social media marketing.

#### Inclusion criteria

Participants must meet all of the following inclusion criteria in order to participate in this study:

1. Female of 16–24 years of age;
2. At least 1 NSSI episode in the 2 months prior to the initiation of the study;
3. At least 5 past episodes of NSSI with significant tissue damage;
4. Psychotropic medications need to be dose-stable for at least 1 month prior to initiation of the study;
5. Ability to understand the study procedures and have willingness at the time of consent to comply with them for the entire length of the study;
6. Depending on the age of the potential participant, either the participant or their parent/guardian (if the participant is a minor) must provide informed consent. Additionally, assent will be obtained and documented from all potential participants of age 16 or 17 years who meet the criteria and are willing to participate.

#### Exclusion criteria

1. Males;
2. Any current serious medical illness as defined by medical history;
  - Current substance use disorder (except tobacco use disorder);
  - Primary psychotic disorder (e.g., schizophrenia, schizoaffective disorder, schizophreniform disorder);
  - Neurodevelopmental disorder such as intellectual disability or autism;
3. Having taken NAC or GSH on a regular basis in the past 6 months;
4. Currently pregnant or breastfeeding, planning to become pregnant, or unwilling to use contraception throughout the study;
5. Allergy/sensitivity to NAC;
6. Inability or unwillingness of individual or legal guardian /parent to give written informed consent.

Additional exclusion criteria related to MR scanning:

1. Any magnetic resonance scanning contraindication (e.g., metal plates, braces, implanted devices, claustrophobia).

#### Sample size calculation

The sample size (*n* = 12 per group) was selected based on the variability estimates from a previous oral NAC pilot study in adults with Parkinson’s disease (*n* = 5) and healthy controls (*n* = 3) [27]. Groups of at least 11 each will have 80% power to show a significant (alpha = 0.0167, Bonferroni corrected for 3 pairwise comparisons) effect of NAC across 3 groups on the primary outcome of brain GSH concentration assuming the variability in this study would be comparable to the pilot study (detectable mean group difference in pre-dose to post-dose percent change of 2.95%, expected mean group difference ~4.83%). We plan to enroll up to 45 participants with the expectation of up to 20% attrition due to participant withdrawal.

#### Interventions

Multiple PBO-controlled clinical trials have shown that oral NAC, typically at modest doses such as 1200 mg–3000 mg/day, produces significant behavioral changes in several psychiatric disorders [9]. However, since NAC pharmacology and pharmacokinetics are complex, neural mechanisms underlying these behavioral effects are still unclear. NAC pharmacokinetics differ substantially depending on the route of administration: while IV doses produce high NAC levels, much of the oral NAC gets metabolized to CYS before reaching systemic circulation, producing lower plasma NAC levels. Further, questions remain about NAC’s central versus peripheral effects. A few clinical studies have demonstrated peripheral measures of antioxidant change on treatment with oral NAC [27,36] but to date there is a paucity of evidence demonstrating that oral NAC leads to significant antioxidant effects in the human CNS. In a small study of patients with Parkinson’s or Gaucher’s disease, our group showed that a single dose of IV NAC led to acute increases in GSH redox ratio in blood (200 fold) and in cortical GSH concentrations (30–50%) as measured by MRS [28]. However, following a 28-day course of high-dose oral NAC (6000 mg/day), antioxidant effects in blood and brain were more modest (6% increase in brain GSH, 2-fold increase in blood GSH redox ratio [GSH/GSSG]) [27]. It is conceivable that a higher NAC dose may be needed to achieve the peripheral and cortical biological changes given low bioavailability of oral NAC, and thus we decided against selecting rather modest NAC doses.

The SW854 NAC product is provided to the M Health Fairview Investigational Drug Service (IDS) pharmacy at the UMN by the manufacturer, Swanson Health Products (ND, USA). SW854 is a gelatin 0-size capsule containing 600 mg NAC. Placebo is manufactured by IDS and matches exactly in appearance with SW854 to achieve blinding. The study medication is dispensed by the IDS in the form of 2 divided daily doses. All participants take 5 capsules of the study medication in the morning and 4 capsules in evening to maintain the blind. The aim of the study is to achieve steady state levels of NAC and its downstream metabolites in order to trigger and sustain the expected biological changes. The dosing interval was selected based on the elimination half-life of NAC which has been reported to be about 6 h [27], and our prior experience documenting favorable patient adherence to twice daily oral NAC dosing [27,33].

#### Study procedures, participant timeline

**Figure 1.**
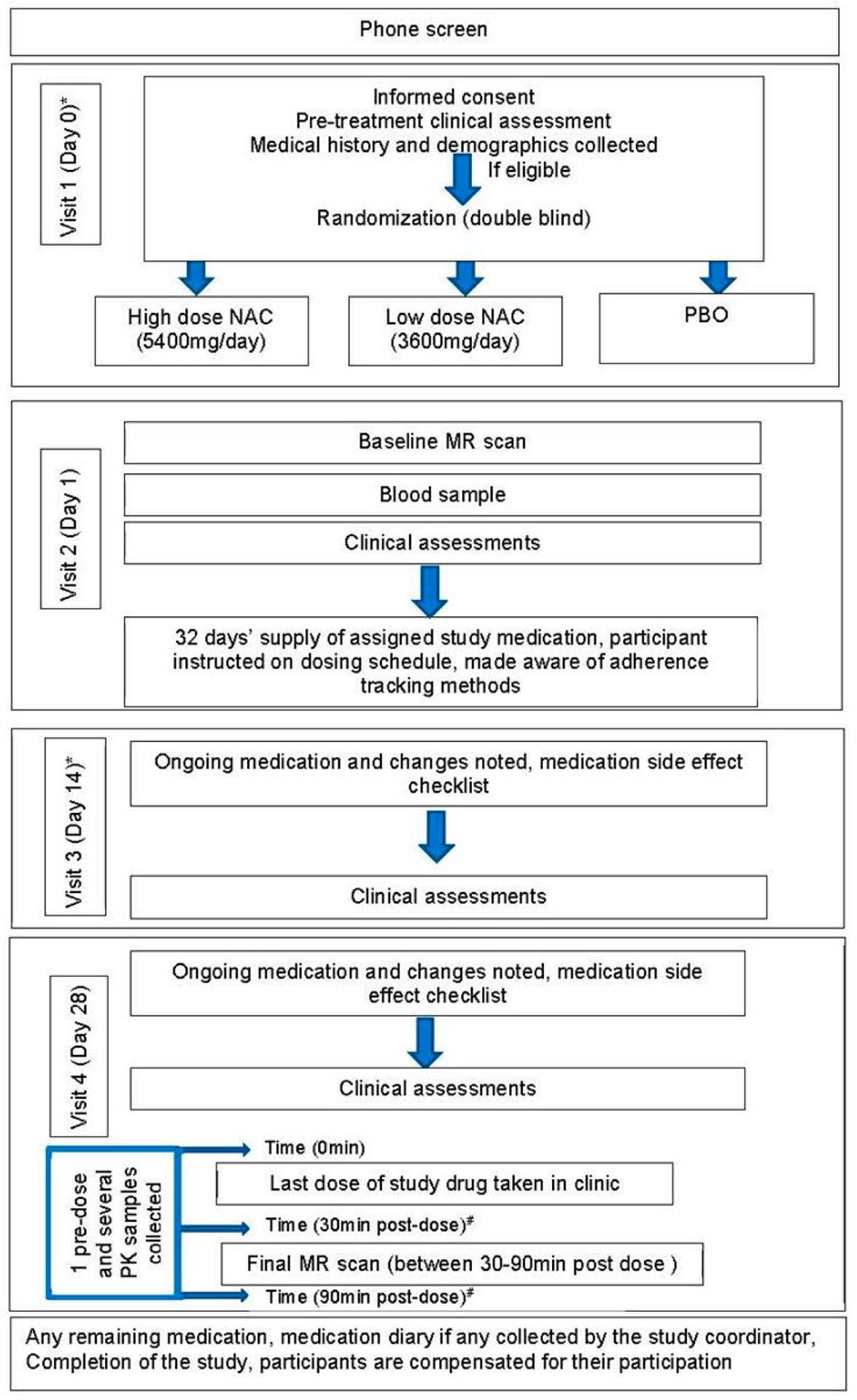
Study outline and participant timeline for the stage 1 (R61). *Visit 1 and 3 can be in-person or through video conference. ^#^ Times are representative.

The stage 1 (R61) study protocol was approved by the Institutional Review Board (IRB) at the UMN and by an external IRB (Advarra IRB), per the clinical research requirements outlined by UMN for the Department of Psychiatry and Behavioral Sciences. In this study there are a total of 4 visits. For 2 of these, patients have the option to complete them through video conferencing. In addition to these visits, there are 2 instances where the participants will electronically complete questionnaires on their own. Eligibility is confirmed by a telephonic screening after which consent is recorded at visit 1. Figure 1 represents the participant timeline. Table 1 details several clinical assessments collected throughout the study.

#### Outcomes

##### Primary outcome

1. GSH concentration in ACC

This measurement will be collected at pre-treatment and post-treatment scanning visits (visit 2 and visit 4 respectively).

##### Secondary outcomes

1. GSH/GSSG in blood collected at visits 2 and 4
2. Glu concentrations in ACC voxel collected at visits 2 and 4
3. Tolerability of NAC as measured using a side effects checklist
4. PK analysis of NAC and its metabolites, CYS and GSH
5. Antioxidant protein levels (catalase and heme oxygenase-1 (HO-1)) collected at visits 2 and 4
6. Gamma-aminobutyric acid (GABA) concentrations in the ACC measured at visits 2 and 4
7. Functional connectivity between amygdala and insula, data collected at visits 2 and 4

#### Clinical assessments

##### Assessment of general psychopathology and demographic information

To describe our sample and to determine eligibility, we will screen for the presence of DSM-5 psychiatric diagnoses [37]. We will conduct a clinical interview with the participant using the MINI scale [38]. Clinicians formulate the diagnosis of substance use disorder combining all clinical information. Recreational substance use is highly pervasive in this age group and is not exclusionary. Furthermore, participants with a diagnosis of substance use disorder may be included in the study if they have had at least 3 months of sobriety. The WASI-II (IQ) [39] will be used to estimate intellectual functioning. The Edinburgh Handedness Inventory [40] will be used to determine handedness. We will monitor the groups’ similarity with respect to socioeconomic status using the Hollingshead Four Factor Index of Social Status [41] after collecting demographic information with a self-report form. We will also collect past antidepressant medication trials with an Anti-Depressant Medication form, other psychotropic medications with Any Psychotropic Medications form and treatment history with Treatment History form, all via the Research Electronic Data Capture (REDCap) software. The CTQ [42] includes 6 questions about past trauma and 7 about recent trauma. Depression severity will be measured using the BDI-II [43] and the PHQ-9 [44]. We will also administer the medication side effects checklist to assess baseline symptom overlap with responses to this checklist at later stages of the study.

##### Assessment of NSSI and Suicidality

We will use the SITBI [45] to record NSSI history (age of onset, total past episodes, episode frequency, reasons for NSSI, thoughts about NSSI, and other information) and use the ISAS-Lifetime to assess the functions and frequency of behaviors of NSSI [46]. We will use the ABUSI scale to assess urges to engage in NSSI. We will use the BSS [47] to assess current suicidal ideation, including active suicidal desire, specific suicide plans, and passive suicidal desire. To retrospectively assess mood before, during and after NSSI events we will use the DSHQ-M. The DTS will be used to assess emotional distress tolerance [48]. We will use the one-question Cash Choice Task as a delay discounting measure to further assess executive functioning [49].

#### MR scan related safety assessments

A urine toxicology screen, urine pregnancy test, and an MR safety screen will be performed at visit 2 and visit 4 before the participant undergoes MR scanning. If a pregnancy test is positive, the participant will not undergo an MR scan and will be removed from the study due to meeting exclusion criteria. If the toxicology screen is positive, results will be noted on the MR case report form and the participant will continue the MR scan. Participants who do not pass the MR safety screen will not receive an MR scan and will be allowed to remain in the study. Results of pregnancy and urine toxicology tests will not be shared with parents/guardians of any participants.

##### MR scanning

All brain scanning will be completed at the CMRR on an actively shielded 7T whole body Siemens MAGNETOM scanner (Siemens Medical Solutions, Erlangen, Germany) using a commercial radiofrequency (RF) head coil of single channel transmit and 32-channel receive (1T×/32R×) produced by Nova Medical (Wilmington, MA, USA). A dielectric pad (180 × 100 × 5.5 mm^3^) made of barium titanate (BaTiO_3_, CAS#12047-27-7) in D_2_O will be placed on the forehead of subjects in order to boost RF signal transmit in the frontal region of the brain.

##### Spectroscopy acquisition and analysis to obtain GSH, GABA and Glu concentrations

Proton spectra will be acquired from the ACC (24 × 30 × 12 mm^3^) using the semi-LASER pulse sequence (TR/TE = 5000/26 ms) [50,51]. Acquisition methods and evaluation of the cerebrospinal fluid (CSF) contribution to the ACC voxel will be performed as described by Terpstra et al. 2016 [52] and Van de Bank et al. 2015 [53].

Anatomical imaging: T_1_-weighted whole brain images will be acquired using 3D MPRAGE sequence (TR/TI/TE = 2890/1500/2.42 ms) at 1.0 × 1.0 × 1.0 mm^3^ resolution. Proton density images will be acquired at 1.0 × 1.0 × 1.0 mm^3^ resolution using 3D MPRAGE sequence (TR/TE = 1410/2.42 ms). Tissue segmentation will be performed on the collected images in order to estimate fractional CSF within the MRS volume-of-interest (VOI) [54]. In addition, the anatomical data will be processed using the Human Connectome Project (HCP) analysis pipeline for segmentation of brain regions.

Based on consensus recommendations [55], only the spectra with associated water reference linewidths of less than 19Hz will be included in the analysis. MATLAB-based MRspa software [56] will be used to perform phase, frequency and eddy-current corrections of channel-combined single-shot spectra before averaging. Averaged MR spectra will be quantified using LCModel with a simulated basis dataset [52] and water scaling option [57,58]. The density-matrix simulated basis set will include aspartate (Asp), glutamine (Gln), glucose (Glc), myo-inositol (Ins), phosphoethanolamine (PE), phosphocholine (PCho), glycerylphosphorylcholine (GPC), creatine (Cr), phosphocreatine (PCr), lactate (Lac), N-acetylaspartate (NAA), N-acetylaspartylglutamate (NAAG) and taurine (Tau), in addition to GABA, Glu and GSH. Brain GSH, Glu and GABA values will be considered for the subsequent analysis only if the across-subject mean of the Cramer-Rao lower bound (CRLB) for each metabolite is equal to or less than 20%.

The MR scanning will start about 30min post dose on the Day 28 (visit 4) and last until about 90min post-dose. Our group has previously reported that the maximum blood concentration of NAC following an oral dose is achieved in ~1.5 h [27]. We needed to balance brain measures and blood measures around this time window to quantitatively capture the maximum likely change in the biological signatures as measured in the brain and the blood.

##### Resting-state functional imaging acquisition and analysis to obtain secondary outcome amygdala-insula resting-state functional connectivity

Resting-state functional data will be acquired using the HCP multiband echo planar imaging sequence for 7T [59,60]. Whole brain T2*-weighted functional volumes (TR/TE = 1000/22.2 ms) will be obtained at 1.6 × 1.6 × 1.6 mm^3^ resolution. Participants will be instructed to keep eyes open while viewing a fixation cross (2 runs at 6 min each). The duration and the choice of fixation cross as the resting condition are selected to optimize reliability [61,62]. Whole-brain functional connectivity maps of the amygdala will be obtained for each person at each time point using methods described previously [63,64].

#### Biological/pharmacological assessments

A single blood sample will be collected at visit 2 for baseline measurements of antioxidant proteins (HO-1, catalase) and glutathione redox status. On visit 4 a pre-dose blood sample will be collected followed by a series of blood samples to characterize NAC and metabolite PK. Following our standard lab procedure, blood will be collected in tubes containing K3EDTA, limiting hemolysis during collection. Plasma and red blood cells will be separated and frozen until analysis. GSH/GSSG and total GSH will be measured in red blood cells using high performance liquid chromatography coupled to a tandem mass spectrometer (HPLC-MS/MS) as previously reported [28,65]. Total (reduced + oxidized) concentrations of NAC and CYS will be measured in plasma using a validated HPLC-MS/MS assay. Plasma HO-1 levels will be determined using HO-1 human ELISA kit (Enzo Life Sciences, Farmingdale, NY) as reported previously [65]. Catalase enzyme activity will be measured in red blood cell lysate using Catalase Assay kit (Cayman Chemical, Ann Arbor, MI) as per manufacturer’s instructions.

According to the original plan, on visit 4 a total of 5 blood samples will be collected immediately following the last dose of NAC up to 6 hours post dose. However, as part of measures taken to accommodate the current need to minimize indoor time spent by the participant in our research facility, an abbreviated blood sample collection scheme will be adopted, that will collect a total of 3 blood samples i.e., one pre-dose, one immediately after dose and one blood sample after the participant finishes their MR scan, cutting down on the visit time. Once the pandemic concerns have passed, we will employ the original sampling scheme, following the review and approval of these changes from the regulatory bodies overseeing this study.

#### Data analysis

##### Primary objective

The within-person percent change in the proposed primary biological signature, brain GSH, will be quantified by fitting a generalized linear model with brain GSH as the dependent variable with group (NAC high dose versus NAC low dose versus placebo) as the predictor variable of interest. Since we apply a minimization procedure to ensure that groups are similar on key demographic and baseline clinical variables, we will not include covariates. Should the primary outcome values be highly skewed, we will use non-parametric tests to compare groups. To account for multiple comparisons, Tukey’s procedure will be used. To assess the robustness of our conclusions about the primary outcome to participants whose medical profile changes during the course of active treatment in our study, we will conduct sensitivity analyses.

##### Secondary objectives

To explore additional potential biosignatures, we will fit generalized linear models with each of the additional outcomes (brain Glu, blood GSH/GSSG, antioxidant protein levels, GABA concentrations, amygdala-insula functional connectivity) as dependent variables, with group (NAC high dose versus NAC low dose versus placebo) as the predictor variable of interest without including covariates. These secondary analyses will use Holm’s step-down Bonferroni type I error correction to adjust for both multiple comparisons among groups and multiple testing across the several secondary outcomes.

NAC PK, NAC and GSH concentration-time data will initially be analyzed by non-compartmental methods. WinNonLin Phoenix (Pharsight^®^) will be used to calculate the partial NAC area under the curve (AUC_0-2 h) using the linear trapezoidal rule and maximum concentration (Cmax). The AUC_0-2h and Cmax for GSH will also be calculated. Descriptive statistics of the PK parameters will be determined. Additionally, the concentration-time data will be analyzed by compartmental methods (Phoenix, Certara). Response markers will be analyzed using established pharmaco-statistical methods designed to detect relationships among these measures and exposure to NAC and/or GSH. Non-linear mixed effects modeling may also be used to develop population-based PK and PK/PD models (NONMEM v. 7.3 or Phoenix NLME, Certara). This approach will allow us to develop a covariate model that includes subject-specific variables (e.g., age, concomitant medications and clinical measures of disease) that demonstrate a significant influence on the PK and/or PD parameters. Drug concentrations and other PK variables will be correlated with degree of change in biological signatures. Changes in blood GSH concentrations as measured in the blood sample collected at the visit 2 and trough or pre-dose blood sample collected at visit 4, will be examined using repeated measures statistical analysis (e.g., repeated measures ANOVA or Friedman’s test for repeated measures). Correlations in brain and blood GSH concentrations will be evaluated using linear regression.

To address the secondary objective of describing the tolerability of oral NAC at the proposed study doses, rates of side effects and other adverse events will be quantified and compared between groups using Poisson, survival, or other rate-based models as appropriate for each event type.

#### Go/No-Go Criteria for Transition from stage 1 to stage 2

Advancement to the stage 2 project will require at least one of the sub-criteria within bullet 1, along with bullets 2, 3, and 4, to hold:

1. Demonstration of change in the proposed biological signatures (GSH and Glu) as defined by:
  - Increased GSH: NAC has the capacity to serve as an antioxidant by increasing GSH, and this is of critical relevance to NSSI. We will examine GSH increase in brain and blood, as follows:
    - GSH concentration in the ACC: We will require a 5% increase in GSH concentrations, as measured by MRS. This change is similar to that observed in healthy controls following 4 weeks of oral NAC (6000 mg/day) [11] and is lower in magnitude than the change observed in patients with Parkinson’s disease and in healthy controls following a single dose of IV NAC 150 mg/kg [12].
    - GSH/GSSG in blood: We will require at least a 100% increase in GSH/GSSG levels. This change will be similar to what we observed in healthy controls following 4 weeks of NAC and is about half of what we observed in patients with Parkinson following 4 weeks of oral NAC 6000 mg/day [11].
  - Glu modulation: NAC has the capacity for modulating the Glu-cysteine antiporter and thereby effectively alleviating excessive glutamatergic neurotransmission and this mechanism is of critical relevance to NSS.
    - Glu concentration in the ACC: To move forward, we will require an 8% decrease in Glu. This change would be similar in magnitude to that seen in patients with cocaine dependence following a single administration of 2400 mg oral NAC [13].
2. Sufficient plasma NAC levels observed in subjects that had been given the dose and showing change in biological signatures as defined above.
3. Evidence that NAC (at the dose meeting Go/No-go criteria for biological signatures described above) is safe (free of severe adverse events) and tolerable (as measured by the side effects form) in adolescents with NSSI.
4. Demonstrated feasibility of recruiting and retaining adolescents with NSSI into a randomized clinical trial, and ability to meet target enrollment according to the proposed timeline.

### Stage 2 (R33)

#### Overview

The stage 2 will seek to replicate our previous findings of NAC versus PBO (dose selected based on stage 1 results) in a longer (8-week) trial with a new sample of adolescents with NSSI. The stage 2 will also measure the correlations among clinical improvement, change in biological signatures and NAC PK.

Sample definition, recruitment strategy, screening, clinical assessment, randomization and blinding will be identical to the stage 1. In addition, methods for MR data acquisition and analysis, blood sample collection/processing/analyses will be identical to the stage 1, except that the final MR scan and the PK studies will be conducted on the final day of the 8-week trial.

#### Study drug administration and frequency of biological assessments

Following informed consent, baseline clinical assessment and (if eligible) randomization, participants will be invited to the CMRR for a baseline visit. Upon arrival, participants will complete a urine toxicology screen and an MRI safety screen, followed by neuroimaging procedures as described for the stage 1. After the baseline scan, participants will be provided with a one-week supply of study drug. All participants will take the same number of identical appearing study medication which will be divided into two daily doses. (NAC dose will be based on the results of the PK-PD analyses done in the stage 1 of the project). Participants will return for clinical assessment visits on days 7, 14, 28 and 42, adherence will be measured through pill count at in-person visits They will meet with the study clinician for a brief medical visit which will involve assessment of side effects, measurement of vital signs, and gathering their general experiences. Participants will complete a modified SITBI to report number of NSSI episodes and urges since last visit and will rate severity of depression using the BDI. They will then receive a supply of study drug to last them until their next appointment. On day 56, participants will be invited to the CMRR for an 8-hour visit. Participants will present their medication boxes for adherence determination. We will place an IV catheter to take a baseline blood sample to measure trough NAC and CYS levels, as well as oxidative stress measurements. Then, participants will take the final dose of the study medication. We will conduct sparse sampling to assess steady-state pharmacokinetics with 6 total blood samples over 6 hours. Repeat neuroimaging will begin 60 min after the final dose and will last 60 min. Following their second scan participants will be interviewed to assess medication side effects using a checklist, and depression symptoms using the modified SITBI and BDI-II.

#### Primary outcome measures

1. The biological signature(s) from the stage 1 study which met milestone criteria.
2. NSSI frequency.
3. Tolerability of NAC over 8 weeks.

#### Secondary outcome measures

1. Biological signatures will be selected based on the results of the stage 1.
2. NSSI urges.

#### Pharmacokinetic analysis

The PK models developed to describe the stage 1 data will be refined to adequately describe the stage 2 data.

#### Statistical analyses

##### Hypothesis 2a.

*NAC (vs. PBO)-related changes in biological signatures as demonstrated in the stage 1 will be* replicated in the context of the longer treatment course. As in the stage 1, we will quantify the percent change in key proposed biological signatures: brain GSH, brain Glu, and blood GSH/GSSG. To test the hypothesis, we will fit general linear models individually for each of brain Glu, brain GSH and blood GSH/GSSG as dependent variables, with group (NAC versus PBO) as the predictor variable. Similar exploratory analyses will be conducted using the secondary outcomes: amygdala-insula resting state functional connectivity, HO-1.

##### Hypothesis 2b.

*Decrease in NSSI frequency will correlate with changes in biological signatures*. Linear models or generalized linear models will be conducted with NSSI change (for frequency: NSSI frequency week 8 divided by NSSI cutting frequency baseline, with a log link; for urges: total ABUSI scores week 8 minus total ABUSI scores baseline) as dependent variables, and the following predictors: biological signature changes (separate analyses for each: ACC GSH change, ACC Glu change, blood GSH/GSSG change), group (NAC versus PBO), and group-by- biological signature interaction. Our primary hypothesis is on the group-specific association of biomarker change with NSSI change. We will also adjust for important demographic and baseline clinical variables (age, Tanner stage, IQ, socio-economic status, baseline depression severity (BDI-II scores), presence of concurrent psychotropic medication, presence of concurrent psychotherapy, trauma history).

##### Hypothesis 2c.

Both extent of change in biological signatures and reduction in NSSI events will correlate with NAC/metabolite exposure. We will explore the association of NAC levels with changes in biological signatures, among those randomized to the NAC group. Specifically, we will use linear or generalized linear regression models to evaluate change in biological signatures and examine whether those with higher NAC levels had larger changes. For these analyses we will adjust for the baseline measures of the biological signatures and baseline NSSI frequency (in addition to other variables listed above).

###### Power:

At this time, we do not have the variability estimates for biological measurements in the target population to conduct a definitive power analysis that would drive our sample size calculation. We are expecting to require a larger sample not for the replication of the biological effects, but because aim 2 involves measuring a correlation between biological change and a decrease in NSSI.

## DISCUSSION

NSSI is a serious and relatively common mental health problem in adolescents, and it is associated with huge personal and societal costs that include increased risk of suicide attempts, death by suicide, hospitalization and disability. To build upon the results reported in our open label, pilot study pointing toward the promise of NAC in alleviating self-injurious behavior, the 2-stage R61/R33 study takes on a neurobiological approach to identify and confirm the probable biological signatures of NAC. In particular, based on the current state of knowledge including our prior work, we are pursuing the hypothesis that key biological changes underlying NAC’s potential efficacy include increasing brain GSH concentrations (enhancing antioxidant effects) and modulating Glu (decreasing stress-induced excitotoxicity). The goal of the stage 1 is to determine these changes (target engagement) and if observed, the next stage i.e., stage 2 is designed to determine if there is a correlation between these changes and clinical improvement (reduction in NSSI frequency).

The proposed oral doses for the stage 1 were selected based on the ease of dosing and incorporating available knowledge on the safe and effective doses of oral NAC reported in the published literature. Our open label, pilot study showed that adolescents with NSSI tolerated oral NAC 3600 mg/day and showed a clinical benefit (reduction in NSSI frequency) after eight weeks [14]. Coles et al recently showed that adults with Parkinson Disease and healthy adults generally tolerated oral NAC 6000 mg/day, with mild to moderate, reversible gastrointestinal adverse effects and showed changes in blood GSH/GSSG [11]. The dose finding stage in this stage 1 study utilizes 3600 mg/day as the low dose and 5400 mg/day as the high dose to identify the optimum dose that is both effective and safe. With the PK information collected in the stage 1 we will examine if there is a relationship between NAC or metabolite exposure and biological signatures. If such relationships are identified, we will be able to use the PK and PD (biological signatures) to construct dose and/or concentration-response models and subsequently perform simulations that will allow us to refine the dosing regimens in the stage 2.

The stage 1 is designed to examine biological changes as opposed to clinical changes. We expect that biological changes due to NAC will be evident prior to clinical change. We are not expecting to observe a significant clinical effect in the short duration of the stage 1, which is why the stage 1 does not include a post-treatment follow-up period. The short treatment duration is designed to reduce subject burden for this early phase where we are focused on the initial test to show change in biological signatures and selecting optimal dose to engage those signatures. On the other hand, we opted against a single-dose strategy, in favor of a 4-week treatment period, to capture measurable changes associated with repeated dosing. The PK and PD characterizations are planned at the end of the protocol, designed to assess drug concentrations once a steady state has been reached, providing enough time to bring about the anticipated biological changes.

We chose a short-echo time (short-TE) MRS protocol, as opposed to edited MRS, to enable simultaneous quantification of all metabolites-of-interest, Glu, GABA and GSH. We are confident in our ability to detect GSH with a short-TE MRS protocol, as prior work has successfully demonstrated the feasibility of detecting biologically relevant changes in GSH using short-TE MRS at 7T [66]. Furthermore, measurement of GSH concentrations in the cerebral cortex using the same semi-LASER protocol has excellent test-retest reproducibility at 7T (coefficients of variance <10%) [52], a critical consideration in our trial design where baseline levels will be compared to post-treatment values. We will also be using automatic VOI prescription using AutoAlign [67] in order to minimize VOI positioning variability between the two sessions.

Some alterations were made to the protocol in the context of the COVID-19 pandemic, in order to balance the need to reduce risks of exposure to the virus with the need to preserve key aspects of the protocol to maintain study integrity and achieve the goals of the study. These modifications were approved by the UMN IRB and the Advarra IRB. During the global COVID-19 pandemic, participants will complete COVID-19 screening prior to all in-person visits. These visits follow pandemic related safety precautions including use of a face mask and social distancing to the extent that can be accommodated given the study procedures. Given that the originally planned duration of visit 4 (~8 h) may introduce risk to participants due to extended time indoors within a shared research facility, we are using an abbreviated PK sampling protocol during the pandemic that collects only half the number of blood PK samples, reducing the visit time by about half (~4 h). This compromised approach still gives us meaningful PK information with the help of pharmacometrics tools to guide dose selection for the next stage. The study PI will make the determination of reverting to the original PK sampling plan for visit 4 once it is safe to do so based on the guidance from the University and the resultant protocol modifications will be reviewed and approved by the IRBs prior to the implementation.

There are some limitations of this design. First, the sample size, especially in the stage 1, is relatively small. The study is powered to detect a significant difference between the 3 groups in the stage 1 on the primary outcome of brain GSH, based on our prior data from NAC/brain GSH studies. However, it is possible that variability in the novel study population differs from that of our prior studies, introducing the risk of being underpowered with a small sample. Second, negative findings in the stage 1 may be attributed to selecting the incorrect dose. We considered including a larger dose-finding component to this study (more than two dose groups). We decided against this approach because it would add significant cost, complexity and subject burden in the early stage of the research. We therefore selected the two doses we deemed most optimal: high enough to feasibly achieve adequate NAC levels that would cause sufficient biological change, and low enough to be tolerable and therefore prevent early drop-out, based on the available evidence as summarized above. Further, given the known variability in NAC PK, our study design will allow us to examine the relationship between NAC or metabolite exposure and biological signatures. This will allow us to refine the dosing regimens in the stage 2. Third, due to the high burden of study activities (e.g., two MR scans, multiple interviews and questionnaires, multiple blood draws), there is a risk of participant drop-out. This pilot study will provide important feasibility data for future larger studies which will inform selection and frequency of measures to include in the design.

With the key innovations such as the use of multi-modal examination of the impact of NAC dosing on the proposed biological signatures and the investigation of the relationship between NAC PK and biological signatures in the clinical population we hope that R61/R33 study will be a critical step forward.

## CONCLUSIONS

The 2-stage R33/R61 mechanism builds in the opportunity to adapt the study design and the outcomes for the subsequent stage of the study based on the accumulating evidence, ensuring milestone-driven, optimized clinical inquiry. These efforts can potentially result in availability of an economic, safe and effective pharmacological agent for treatment of patients with NSSI.

## Figures and Tables

**Table 1.** Schedule of events and assessments.

Measure/Activity/Event	Day 0 Visit 1*	Day 1 Visit 2	Day 7	Day14 Visit 3*	Day 21	Day 28 Visit 4
Informed Consent Form (HIPAA/Consent/Assent)	×					
Mini International Neuropsychiatric Interview (MINI)	×					
Wechsler Abbreviated Scale for Intelligence-II (WASI-II)		×				
Edinburgh Handedness Inventory	×					
Demographics Form	×					
Childhood Trauma Questionnaire (CTQ)	×					
Antidepressant Medications	×					
Any Psychotropic Medications	×					
Treatment History	×					
Inventory of Statements About Self-Injury-Lifetime (ISAS-Lifetime)	×					
Distress Tolerance Scale (DTS)	×					
Beck Scale for Suicidal Ideation (BSS)	×	×				×
Patient Health Questionnaire (PHQ-9)		×	×	×	×	×
Ongoing Medication Use and Changes: Initial Visit		×				
Ongoing Medication Use and Changes: Subsequent Visit			×	×	×	×
Alexian Brothers Urge to Self-Injure (ABUSI)		×				×
Beck Depression Inventory (BDI-II)	×					×
Self-Injurious Thoughts and Behaviors Interview (SITBI)		×				×
Inventory of Statements About Self-Injury- Since Last Visit (ISAS-SLV)		×	×	×	×	×
Deliberate Self-Harm Questionnaire, Part III Mood (DSHQ-M)		×				×
Cash Choice Task		×				×
Medication Side Effect Checklist		×	×	×	×	×
Magnetic Resonance (MR) Safety Screen, Urine Toxicology Screen, Pregnancy Test		×				×
MRI, MRS, rs-fMRI, Blood Sample (biomarkers)		×				×
Serial blood samples (PK)						×

* Visits 1 and 3 can be in-person or through video conference.
